# Novel Inhibitors of *Staphylococcus aureus* Virulence Gene Expression and Biofilm Formation

**DOI:** 10.1371/journal.pone.0047255

**Published:** 2012-10-15

**Authors:** Yibao Ma, Yuanxi Xu, Bryan D. Yestrepsky, Roderick J. Sorenson, Meng Chen, Scott D. Larsen, Hongmin Sun

**Affiliations:** 1 Department of Internal Medicine, University of Missouri, Columbia, Missouri, United States of America; 2 Vahlteich Medicinal Chemistry Core, College of Pharmacy, University of Michigan, Ann Arbor, Michigan, United States of America; 3 Nanova, Inc., Columbia, Missouri, United States of America; Charité-University Medicine Berlin, Germany

## Abstract

*Staphylococcus aureus* is a major human pathogen and one of the more prominent pathogens causing biofilm related infections in clinic. Antibiotic resistance in *S. aureus* such as methicillin resistance is approaching an epidemic level. Antibiotic resistance is widespread among major human pathogens and poses a serious problem for public health. Conventional antibiotics are either bacteriostatic or bacteriocidal, leading to strong selection for antibiotic resistant pathogens. An alternative approach of inhibiting pathogen virulence without inhibiting bacterial growth may minimize the selection pressure for resistance. In previous studies, we identified a chemical series of low molecular weight compounds capable of inhibiting group A streptococcus virulence following this alternative anti-microbial approach. In the current study, we demonstrated that two analogs of this class of novel anti-virulence compounds also inhibited virulence gene expression of *S. aureus* and exhibited an inhibitory effect on *S. aureus* biofilm formation. This class of anti-virulence compounds could be a starting point for development of novel anti-microbial agents against *S. aureus*.

## Introduction

*Staphylococcus aureus* is a major human pathogen that causes skin, soft tissue, respiratory, bone, joint and endovascular infections, including life-threatening cases of bacteremia, endocarditis, sepsis and toxic shock syndrome [1]. Approximately 30% of humans are *Staphylococcus aureus* carriers without symptoms [2].

*S. aureus* is also one of the most common pathogens in biofilm related infections of indwelling medical devices which are responsible for billions in healthcare cost each year in the United States [3]–[8]. Bacteria can attach to the surface of biomaterials or tissues and form a multilayered structure consisting of bacterial cells enclosed in an extracellular polymeric matrix [9]. Bacteria in biofilm are particularly resistant to antibiotic treatment [10]. In addition to the difficulty of effectively inhibiting biofilm with conventional antibiotic therapy, treatment is further complicated by the rise of antibiotic resistance among staphylococci. In recent years, methicillin resistance in *S. aureus* is approaching an epidemic level [2], [11]–[13].

The emergence of antibiotic resistance poses an urgent medical problem worldwide. Current antibiotics target a small set of proteins essential for bacterial survival. As a result, antibiotic resistant strains are subjected to a strong positive selection pressure. Inappropriate and excessive use of antibiotics have contributed to the emergence of pathogens that are highly resistant to most currently available antibiotics [14]–[16]. The novel approach of inhibiting pathogen virulence while minimizing the selection pressure for resistance holds great promise as an alternative to traditional antibiotic treatment [17]. The feasibility of such an approach was demonstrated for *Vibrio cholerae* infections when a novel small molecule was identified that prevented the production of two critical virulence factors, cholera toxin and the toxin coregulated pilus. Administration of this compound *in vivo* protected infant mice from *V. cholerae* [18]. In a similar proof-of-concept (POC) study, a small molecule inhibitor of the membrane-embedded sensor histidine kinase QseC was identified. The inhibitor exhibited *in vivo* protection of mice against infection by *Salmonella typhimurium* and *Francisella tularensis* [19].

In a POC study following the same paradigm, we have identified a chemical series of small molecules from a high throughput screen (HTS) that can inhibit expression of the streptokinase (SK) gene in group A streptococcus (GAS) [20]. We previously demonstrated that SK is a key virulence factor for GAS infection [21]. SK activates human plasminogen into an active serine protease that degrades fibrin, a critical component of blood clots and an important line of defense against bacterial pathogens [22], [23] Our novel SK gene expression inhibitor also inhibited gene expression of a number of important virulence factors in GAS. The lead compound demonstrated *in vivo* efficacy at protecting mice against GAS infection, further supporting the feasibility of this novel anti-virulence approach to antibiotic discovery [20].

We subsequently expanded our work on the novel antimicrobial agents in GAS to *S. aureus* and demonstrated that this class of compounds is capable of inhibiting *S. aureus* virulence, especially biofilm formation.

## Results

### Identification of Small Molecules Inhibiting *Staphylococcus aureus* Biofilm Formation

**Figure 1:**
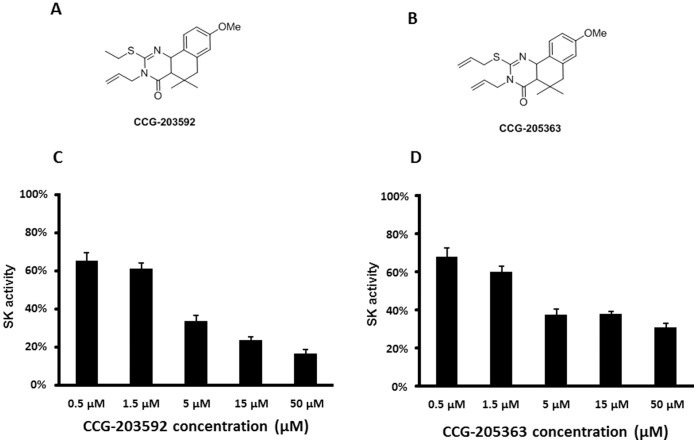
Compound structures and effects on SK expression. A) Structure of CCG-203592 B) Structure of CCG-205363 C) Effects of CCG-203592 on the production of SK activity. Normalized SK activity of GAS treated with CCG-203592 at concentrations from 0.5 to 50 µM (SK activity of culture media divided by OD_600 nm_ of bacteria culture, then normalized to the value for DMSO treated GAS which was defined as 100%). The data is presented as mean±standard error of means for a total of 9 samples (pooled from 3 independent experiments in triplicate). D) Effect of CCG-205363 on the production of SK activity. The value was presented as mean±standard error of means for a total of 9 samples (pooled from 3 independent experiments in triplicate).

Sixty eight novel analogs of HTS lead GAS SK expression inhibitor CCG-2979 [20] were synthesized and demonstrated inhibitory effect on SK expression (manuscript in preparation). These compounds were tested for their effects on *S. aureus* Newman biofilm formation in polystyrene microtiter plates by the standard crystal violet staining method [24]. Two of these analogs, CCG-203592 and CCG-205363 (Figure 1A and 1B), demonstrated reproducible inhibition of biofilm formation. CCG-203592 reduced biofilm formation by 45.2±3.9% and CCG-205363 reduced biofilm formation by 27.8±8.1% at 20 µM.

**Figure 2:**
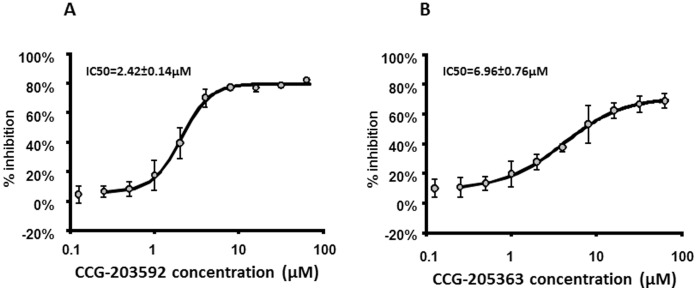
The effect of CCG-203592 and CCG-205363 on *S. aureus* biofilm formation. A) Dose-response curve of CCG-203592 inhibition on RN6390 biofilm formation. The data is presented as % inhibition mean±standard error of means for a total of 9 samples (pooled from 3 independent experiments in triplicate). Percent inhibition is relative to DMSO control. B) Dose-response curve of CCG-205363 inhibition on RN6390 biofilm formation. The value was presented as % inhibition mean±standard error of means for a total of 9 samples (pooled from 3 independent experiments in triplicate).

Both CCG-203592 and CCG-205363 had demonstrated more potency than their lead compound CCG-2979 at inhibiting SK expression (Figure 1C and 1D) [20]. The effect of CCG-203592 and CCG-205363 on biofilm formation was further tested with *S. aureus* RN6390 strain which is widely used for studying biofilm formation [25], [26]. RN6390 was treated with different concentrations of CCG-203592 and CCG-205363, and biofilm formation was measured to estimate the IC_50_s of the compounds. Both demonstrated encouraging inhibition potency with IC_50_ = 2.42±0.14 µM for CCG-203592 (Figure 2A) and IC_50_ = 6.96±0.76 µM for CCG-205363 (Figure 2B). The more potent CCG-203592 was chosen for further analysis.

**Figure 3:**
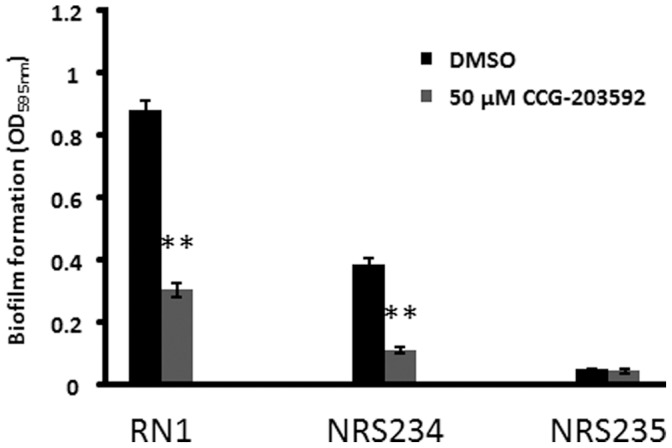
The effect of 50 µM CCG-203592 on *S. aureus* RN1, NRS234 and NRS235 biofilm formation. Biofilm formation was determined by OD_595 _nm reading of crystal violet stain solubilized by ethanol with DMSO treatment as controls. The data is presented as mean±standard error of means for a total of 9 samples (pooled from 3 independent experiments in triplicate). ** p<0.01.

The effect of CCG-203592 on *S. aureus* biofilm formation was further tested with more relevant clinical strains. RN6390 was derived from *S. aureus* RN1 that was originally isolated from a sepsis patient [27]. NRS234 and NRS235 are clinical isolates associated with native valve endocarditis in the Network on Antimicrobial Resistance in Staphylococcus aureus program (NARSA) collection. Native valve endocarditis is strongly associated with biofilms [28]. As a result, these clinical strains were used to test the anti-biofilm effect of CCG-203592. There was little biofilm formation by NRS235 while significant biofilm formation was observed with RN1 and NRS234 (Figure 3). Fifty µM CCG-203592 was able to inhibit biofilm formation of RN1 (65.4±2.3%) and NRS234 (70.2±3.6%) significantly (p<0.001).

### Validation of *S. aureus* Biofilm Inhibition on Silicone Surface

**Figure 4:**
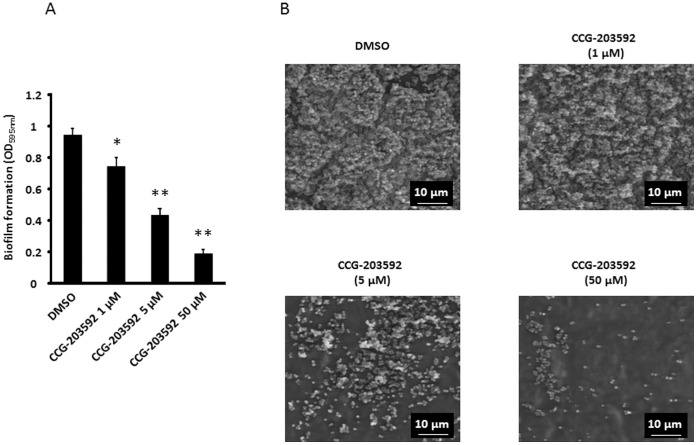
The effect of CCG-203592 on *S. aureus* biofilm formation on silicone wafer. A) RN6390 biofilm formation on medical grade silicone wafer at different concentrations as determined by OD_595 _nm reading of crystal violet stain solubilized by ethanol. The data is presented as mean±standard error of means for a total of 9 samples (pooled from 3 independent experiments in triplicate). * p<0.05, ** p<0.01. B) Scan electron microscopy representative images of RN6390 biofilm formation on silicone wafer treated with different concentrations of CCG-203592 from triplicate.

In order to further characterize the effect of CCG-203592 on *S. aureus* biofilm formation, RN6390 was treated with different concentrations of CCG-203592 and biofilm formation on medical grade silicone was measured. Medical grade silicone is widely used in implantable medical devices [29]. A dose-dependent inhibition of biofilm formation by CCG-203592 was also observed (Figure 4A). The minimum concentration at which significant inhibition was observed (1 µM, p<0.02) is similar to what was observed with the polystyrene microtiter plate assays.

Scanning electron microscopy (SEM) analysis was carried out to visualize the detailed architecture of biofilm. Bacterial cells on control wafers formed multilayered conglomerated clusters with numerous bacterial cells (Figure 4B). At the lowest concentration of CCG-203592 (1 µM), the silicone surface was covered with multilayered dense clusters of bacterial cells, similar to control samples. However, at 5 µM CCG-203592, the biofilm structure was disrupted and a significant part of the silicone surface was cleared of bacterial cells. Bacterial cell clusters were much less dense than that of control biofilm. At 50 µM CCG-203592, there were only small clusters of cells scattered on the surface.

### Toxicity of CCG-203592 in *S. aureus* and Mammalian Cells

**Figure 5:**
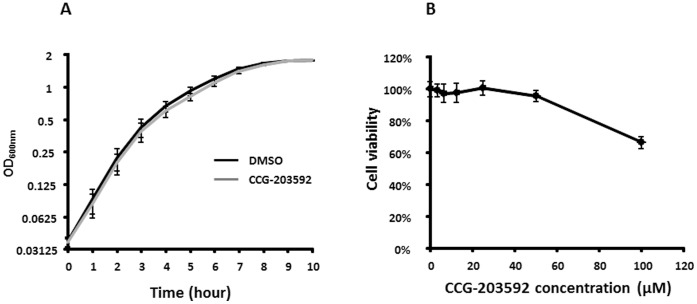
The effect of CCG-203592 on *S. aureus* and mammalian cell viability. A) Growth curves for RN6390 in the presence of CCG-203592 (50 µM) (grey curve) or DMSO alone (dark curve) as determined by OD_600 nm_. The data is presented as mean±standard error of means for a total of 9 samples (pooled from 3 independent experiments in triplicate). B) HeLa cell viability (as determined by mitochondrial reduction of MTT substrate) in the presence of CCG-203592 at different concentrations normalized to the value for DMSO treated samples which was defined as 100%. The data is presented as mean±standard error of means for a total of 12 samples (pooled from 3 independent experiments in quadruplicate).

The chemical series of compounds to which CCG-203592 belongs was developed as a class of novel anti-virulence agents that can inhibit bacterial virulence without inhibiting bacterial growth in order to minimize the chance of developing resistance [20]. The effect of CCG-203592 on *S. aureus* RN6390 growth was therefore tested. Growth of RN6390 in the presence of 50 µM CCG-203592 was analyzed over a period of 10 hours. No significant difference was detected when bacteria were treated with CCG-203592 compared to DMSO control (Figure 5A).

Cytotoxicity of CCG-203592 to mammalian cells was also tested on HeLa cells. The cytotoxicity of CCG-203592 at concentrations of 3.125, 6.25, 12.5, 25, 50 and 100 µM was determined by the colorimetric MTT viability assay and compared to cell viability when treated with DMSO control (Figure 5B). Dose-response studies revealed that no significant cytotoxicity was detected by CCG-203592 up to a concentration of 50 µM (p>0.46). At 100 µM, CCG-203592 displayed cytotoxic activity on HeLa cells and the cell survival rate was 66.4±3.9% (p<0.001).

### Inhibition of Gene Expression of *S. aureus* Virulence Factors by CCG-203592

**Figure 6:**
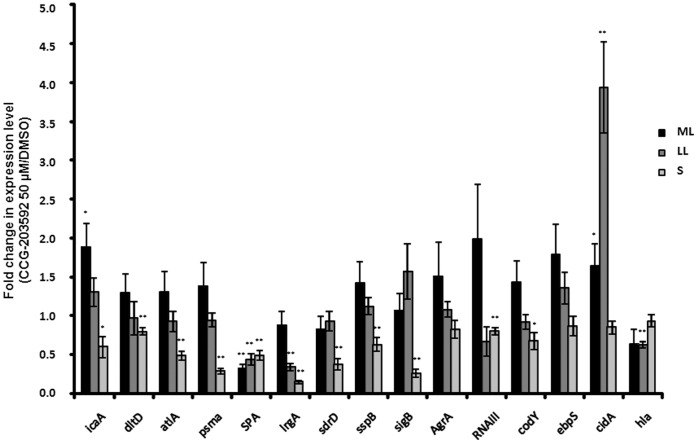
The effect of CCG-203592 on expression of selected *S. aureus* genes. Real time RT-PCRs were performed at mid-logarithmic growth phase (ML), late logarithmic growth phase (LL) and stationary (S) phase. The values are presented as the fold of change of gene transcriptional level of samples treated with 50 µM CCG-203592 versus that of samples treated with DMSO as calculated by 2^(−ΔΔ Ct)^ method. The data is presented as mean±standard error of means for a total of 9 samples (pooled from 3 independent experiments in triplicate). * p<0.05, ** p<0.01.

The chemical series of compounds represented by CCG-203592 was shown to inhibit gene expression in GAS [20]. As a result, we hypothesized that CCG-203592 might also inhibit gene expression in *S. aureus.* A number of *S. aureus* RN6390 genes were selected that were reported to play roles in *S. aureus* virulence and biofilm formation (Table 1). Among the 15 genes tested, *AgrA* and *ebps* demonstrated no significant changes of gene expression when treated with 50 µM CCG-203592 (Figure 6).

**Table 1:** Virulence factor genes tested by Real time RT-PCR.

Gene	Function	Primers	Reference
16S rRNA	Internal standard gene	F: CTGGTAGTCCACGCCGTAAAC R: CAGGCGGAGTGCTTAATGC	[71]
icaA	Polysaccharide intercellular adhesion/polymeric N-acetyglucosamine production	F: AACAGAGGTAAAGCCAACGCACTC R: CGATAGTATCTGCATCCAAGCAC	[35]
dltD	Esterification of teichoic acids with D-alanine	F: GTGCTGCTGGTGCAGATGTTCAAT R: CTGCTTGACGACGTTCTTTATCG	[31], [36]
atlA	Autolysin	F: TGTCGAAGTATTTGCCGACTTCGC R: TGGAATCCTGCACATCCAGGAAC	[32], [37]
Psmα operon	Phenol soluble modulins α	F: ACCCATGTGAAAGACCTCCTTTGT R: ATGGGTATCATCGCTGGCATC	[52], [72]
SPA	Surface and secreted protein for bacterial aggregation	F: GCGCAACACGATGAAGCTCAACAA R: ACGTTAGCACTTTGGCTTGGATCA	[38]
lrgA	Cell death and lysis	F: CTGGTGCTGTTAAGTTAGGCGAAG R: GGCTGGTACGAAGAGTAAGCCAAT	[53]
sdrD	SD-repeat-containing protein	F : AGTACACAGTGGGAACAGCATC R : TCTGCAGCCTTTGCTTCTTGGTTC	[41], [42]
sspB	Cysteine protease	F: CCAGCAAATTGTTGTTGTGCTAG R: AAGCCAAAGCCGATTCACACTC	[44], [73]
SigB	Gene expression regulator	F: TCAGCGGTTAGTTCATCGCTCACT R: GTCCTTTGAACGGAAGTTTGAAGCC	[46]
AgrA	Gene expression regulator	F: AAGCATGACCCAGTTGGTAACA R:ATCCATCGCTGCAACTTTGTAGA	[74]
RNAIII	Gene expression regulator	F: GCACTGAGTCCAAGGAAACTAACTCT R: AGCCATCCCAACTTAATAACCATGT	[46]
CodY	Gene expression regulator	F: AAAGAAGCGCGCGATAAAGCTG R: TGCGATTAATAGGCCTTCCGTACC	[55], [56]
ebpS	Surface protein	F: TTTCCGGTGAACCTGAACCGTAGT R: ACAGCAACAACAACGTCAAGGTGG	[42]
cidA	Cell death and lysis	F: AGCGTAATTTCGGAAGCAACATCC R: TACCGCTAACTTGGGTAGAAGACG	[54]
Hla	Alpha-Toxin	F: CTGAAGGCCAGGCTAAACCACTTT R: GAACGAAAGGTACCATTGCTGGTCA	[51]

Other genes demonstrated significant changes of gene expression by CCG-203592 (Figure 6). Expression of *icaA* gene of the *icaADBC* operon that is responsible for polysaccharide intercellular adhesin (PIA) or polymeric N-acetyl-glucosamine (PNAG) production [30] was increased by 88.3±30.2% (p<0.02) at mid-logarithmic (ML) growth phase and decreased by 40.2±13% (p<0.05) at stationary (S) phase compared to control by treatment with 50 µM CCG-203592. Expression of *dltD* gene of the *dlt* operon that is involved in D-alanine incorporation into teichoic acids [31] was decreased by 20.6±4.5% (p<0.01) at S phase. Bifunctional autolysin *atl* gene expression [32] was reduced by 51.7±5.6% (p<0.01) at S phase. Expression of *psmα* operon which produces phenol soluble modulins α (PSMα1–4) peptides [33] was decreased by 71.6±3.8% (p<0.01) at S phase. Immunoglobulin G-binding protein A (SPA) gene expression was decreased by 68.1±6.1% (p<0.01) at ML phase, 56.2±7.2% (p<0.01) at late logarithmic (LL) growth phase and decreased by 50.6±6.2% (p<0.01) at S phase. Murein hydrolase regulator *lrgA* gene expression was decreased by 66.7±5.1% (p<0.01) at LL phase and decreased by 85.6±2.3% (p<0.01) at S phase. SD-repeat-containing protein D (sdrD) gene expression was reduced by 62.6±7.2% (p<0.01) at S phase. Cysteine protease (sspB) gene expression was decreased by 37.4±9.1% (p<0.01) at S phase. Sigma B (*SigB*) gene expression was decreased by 73.5±5.1% (p<0.01) at S phase. RNAIII gene expression was reduced by 20.1±4.5% (p<0.01) at S phase. Gene repressor CodY expression was reduced by 32.7±10.9% (p<0.05) at S phase. The murein hydrolysin regulator *cidA* gene expression was increased by 65.1±27.2% (p<0.05) at ML phase and 294.0±58.7% (p<0.01) at LL phase. The alpha-toxin (*Hla*) gene expression was decreased by 37.1±3.8% (p<0.01) at LL phase.

## Discussion

In recent years, antibiotic resistance has become one of the biggest threats to public health. Conventional antibiotics aim to kill or inhibit the growth of bacteria, leading to a strong selective advantage for resistant pathogens. As a result, a new approach to developing antimicrobial agents has been proposed that entails targeting virulence of the pathogens without inhibiting their growth, thereby reducing or slowing the selection for resistance [17]–[20], [34].

In our previous studies, we identified a novel chemical series of low molecular weight compounds that can inhibit expression of group A streptococcus virulence gene expression, leading to *in vivo* efficacy at protecting mice against GAS infection [20]. These compounds demonstrated little interference with GAS growth following the new approach above to develop novel antimicrobial agents [17]–[19], [34]. In order to further improve the potency and pharmacokinetic properties of this class of anti-virulence compounds, we have been carrying out Structure Activity Relationship (SAR) studies by synthesizing and characterizing more compounds in this chemical series (manuscript in preparation). In an effort to test whether these anti-virulence compounds have broad spectrum efficacy against other gram positive pathogens, we tested their effects on *S. aureus* biofilm formation.

A total of 68 compounds (those that were active against GAS SK expression) from the SAR program were tested for effects on biofilm formation of *S. aureus* Newman strain. Two of the compounds, CCG-203592 and CCG-205363, demonstrated consistent inhibition of biofilm formation. These two compounds were further tested for their potency at inhibiting biofilm formation using the widely studied biofilm strain, RN6390. Both compounds demonstrated significant inhibition potency with IC_50_s in the low micromolar range.

Further studies with the more potent compound CCG-203592 also showed that the compound can inhibit biofilm formation of clinically associated strain RN1 and NRS234 and also inhibit biofilm formation on the surface of medical grade silicone which is widely used in medical devices such as catheters that are particularly prone to *S. aureus* biofilm-related infection [3], [29]. Scanning electron microscopy analysis of biofilm on the surface of silicone wafers indicated that CCG-203592 was able to disrupt the biofilm structure. At higher concentrations (≥5 µM), it actually prevented colonization of bacteria on major areas of the silicone surface.

The effect of CCG-203592 on *S. aureus* growth was also studied. CCG-203592 had no effect on bacterial growth, which is similar to its analogs’ lack of growth inhibition of GAS [20]. The cytotoxicity of CCG-203592 was also tested with HeLa cells. Human HeLa cells demonstrated good tolerance to treatment with CCG-203592. The result suggested that CCG-203592 has minimal to no cytotoxicity at a concentration (50 µM) that can inhibit 80% biofilm formation and also significantly inhibit the expression of a number of virulence factor genes.

The lead compound of this class of anti-virulence compounds was identified as a repressor of SK gene expression in GAS, and a structurally related analog altered gene expression of a number of virulence factors in GAS [20]. We thus hypothesized that CCG-203592 could also change gene expression of *S. aureus* virulence factors. Biofilm formation proceeds through multiple steps involving the initial attachment step in which bacterial cells bind to the surface, a maturation step in which bacteria will accumulate and proliferate on the surface to form mature biofilm structures and finally detachment of bacterial cells for dissemination to other colonization sites [7]. A number of genes have been reported to be involved in these steps of biofilm formation. Some of these genes were selected for evaluation of their susceptibility to gene expression inhibition by CCG-203592 using a real time RT-PCR approach.

The genes down-regulated or up-regulated by CCG-203592 are involved in biofilm formation at different stages of biofilm formation. The *icaADBC* operon encodes enzymes involved in biosynthesis of polysaccharide intercellular adhesin (PIA) or polymeric N-acetyl-glucosamine (PNAG) that plays important roles in biofilm formation [30]. Deletion of the *ica* locus significantly decreased *S. aureus* biofilm formation [35]. Down-regulation of *icaA* could decrease production of PIA/PNAG, leading to reduction of biofilm formation. Interestingly, *icaA* was up-regulated during ML phase, but down-regulated at S phase. The net outcome of the effect of CCG-203592 on *icaA* could result from the combined effect of the dynamic changes of gene expression.

The *dltABCD* operon encodes four proteins responsible for esterification of teichoic acids with D-alanine [31]. Deficiency in dltA results in a stronger negative net charge on the bacterial cell surface and defects in the initial binding of bacteria to the surface in biofilm formation [36]. Down-regulation of *dltD* in the same operon could have similar effects. Autolysin altA is a major peptidoglycan hydrolase that cleaves newly synthesized peptidoglycan components before they are incorporated into the cell wall [32]. Primary attachment of bacteria to surfaces is impaired in *altA* null mutants [32], [37]. SPA gene was consistently down-regulated by CCG-203592 in all three phases tested. SPA is able to induce cell aggregation and biofilm formation [38]. sdrD is one of the microbial surface components recognizing adhesive matrix molecules (MSCRAMM) that play important roles in mediating bacteria adhesion to host tissues and forming biofilm though the exact function of sdrD is unkown [39]–[42]. *sspB* encodes a cysteine protease that is regulated by *agr* system [43]. Inactivating *sspC* which is an inhibitor of sspB, enhances the attachment of bacteria to solid surfaces and biofilm formation, suggesting that sspB has positive effects on biofilm formation [44].

SigB is an alternative sigma factor that regulates a large regulon [45] and inactivating *SigB* decreases biofilm formation by *S. aureus* and increases RNAIII level [46]. RNAIII is a component of the *agr* quorum-sensing system which regulates gene expression in response to outside signals [47]. Inhibition of *agr* system is important for biofilm development and *agr* also mediates biofilm dispersal [48], [49]. The influence of *agr* system on biofilm development is multifaceted and complicated, depending on experimental conditions [50]. Hla was shown to be required for *S. aureus* biofilm formation and deficiency in Hla caused defects in biofilm formation [51]. Taken together, down-regulating the above genes could negatively impact biofilm formation.

On the other hand, *psmα* operon encodes four short PSMα peptides (∼20 amino acids) [33]. Deletion of *psmα* causes defects in formation of biofilm channels and biofilm detachment and regrowth which suggested that PSMs are important for biofilm maturation and detachment. Lack of PSMs led to increased biofilm volume and thickness [52]. The *lrg* operon is responsible for inhibition of murein hydrolase activity of the CidA protein. Mutant inactivating *LrgAB* operon exhibits increased biofilm adherence and matrix-associated eDNA, and forms biofilm with reduced biomass and defective structures compared to mature wild-type biofilm [53]. Interestingly, *CidA* was up-regulated during ML and LL phases which could generate similar phenotype as down-regulating *lrg* [54]. However, mutations in both *lrg* and *CidA* caused aberrant biofilm maturation, suggesting that imbalance in their gene expression could disrupt biofilm development [53]. These effects of CCG-203592 may increase biofilm formation, which could be outweighed by the effects of down-regulation of other genes by CCG-203592. As a result, the combined effect of all the affected genes by CCG-203592 may produce net decrease of biofilm formation.

Interestingly, CCG-203592 decreased the RNAIII level slightly, suggesting that up-regulation of RNAIII level by decreased SigB and CodY level was compensated by changes in other genes that may also regulate RNAIII level. CodY is another global gene regulator that represses *agr* and *icaADBC* operon [55]. Inhibition of *CodY* could have different effects on biofilm formation. Inactivating *CodY* could enhance biofilm formation in *S. aurues* strain SA564 and UAMS-1 [55], but reduce biofilm formation in high-biofilm-producing *S. arueus* isolate S30 [56].

More genes were affected by CCG-203592 at stationary phase than at growing phases. We also observed that an analog of CCG-203592 changed expression of more genes at stationary phase than at growing phases in GAS [20]. It was well known that expression patterns of many genes are changed at different growth phases. For example, depletion of glucose and change of pH after a long period of culture at stationary phase could impact the gene expression of *agr* system [57]. As a result, it is possible that CCG-203592 has different impacts on gene expression at different growth phases. In order to understand the mechanism of action of this novel anti-virulence compound, further studies on the impact of gene expression changes at different growth phases on biofilm formation are needed.

Of note, some of the genes that have been down-regulated also play important roles in staphylococcus virulence. SPA, Hla and PSMs are virulence factors [33], [58]–[62] and sspB plays important roles in staphylococcus evasion and resistance to host defense [63]. Based on the gene profile changes by CCG-203592, down-regulation of these genes could lead to defects in biofilm formation at different stages and could also lead to diminished virulence.

In conclusion, this class of novel anti-virulence compounds demonstrates inhibitory effects on gene expression of multiple *S. aureus* virulence factors, especially genes known to be involved in biofilm formation, resulting in significant inhibition of biofilm formation. The compounds also inhibit SK gene expression in GAS, suggesting that this class of compounds could target a gene regulatory mechanism that is conserved between GAS and *S. aureus*. This class of compounds could be a starting point for development of novel anti-microbial agents against multiple pathogens.

## Materials and Methods

### Bacterial Strains and Culture Conditions

GAS strain UMAA2616 was derived from the M type 1 strain MGAS166 [64]. The laboratory bacterial strains *S. aureus* Newman and RN6390 were used in this study. *S. aureus* Newman, a human clinical strain isolated from a case of secondarily infected tubercular osteomyelitis [65], was kindly provided by Dr. Olaf Schneewind, University of Chicago. *S. aureus* RN6390 [66] is a strain derived from RN1 [67] which was isolated from a sepsis patient. *S. aureus* RN6390 were provided by NARSA, which is supported under NIAID, NIH Contract No. HHSN272200700055C. NRS234 and NRS235 are clinical isolates associated with native valve endocarditis from NARSA.

The UMAA2616 strain was grown in Todd-Hewitt broth containing 0.2% yeast extract (THY) (Difco, Detroit, MI) supplemented with 100 µg/mL streptomycin [21]. Planktonic culture of *S. aureus* was grown in THY. The medium for growth of static biofilms was THY with 0.5% glucose. All bacterial cultures were incubated at 37°C.

### Synthesis of CCG-2979 Analogs

CCG-203592 and CCG-205363 were synthesized in the Vahlteich Medicinal Chemistry Core laboratory at the University of Michigan. The procedures will be described in a separate publication (manuscript in progress).

### SK Activity Assay

The SK activity assay was described previously [20]. Briefly, overnight UMAA2616 culture was diluted 1∶1000 into fresh THY medium containing different concentrations of CCG-203592, CCG-205363 or DMSO and grown at 37°C to an OD_600 nm_ = 1.0 in triplicate. Twenty µl of culture supernatant was added to 100 µl phosphate buffered saline (PBS), 10 µl human plasma (Innovative Research, Novi, MI), and10 µl S2403 (1 mg/ml) (Diapharma group Inc., West Chester, OH) and incubated at 37°C for 2 hours. SK activity was measured by OD_405__nm_ and calculated as the percentage of SK activity compared to a DMSO control UMAA2616 culture. The experiment was performed three times to obtain the mean and standard error of means for SK activity of each treatment.

### Biofilm Assay

The biofilm assay was performed using 96-well polystyrene flat-bottom microtiter plate (TPP Techno Plastic Products AG, Trasadingen, Switzerland) or on medical-grade silicone wafers (Cardiovascular Instrument Corp. Wakefield, MA). Silicone wafers were placed in wells of 12-well polystyrene flat-bottom microtiter plate (TPP Techno Plastic Products AG, Trasadingen, Switzerland).

Overnight cultures of *S. aureus* were diluted 1∶200 with fresh sterile THY medium containing 0.5% glucose. For the screening of test compounds, wells of 96-well microtiter plates were filled with 200 µl aliquots of the diluted cultures with 20 µM CCG-2979 analogs or DMSO in triplicate. For estimating IC_50_ (the half maximal inhibitory concentration), wells of 96-well microtiter plates were filled with 200 µl aliquots of the diluted cultures with different concentrations of CCG-203592, CCG-205363 or DMSO in triplicate. For testing the effect of CCG-203592 on biofilm formation of strains RN1, NRS234 and NRS 235, 50 µM CCG-203592 was used. For measuring biofilm formation on silicone wafers (1×1 cm), wells of 12-well microtiter plate containing silicone wafers were filled with 1 ml aliquots of the bacterial culture with different concentrations of CCG-203592 or DMSO in triplicate. The plates were incubated overnight at 37°C [50], [68]. Non-adherent bacteria were washed by PBS for three times. Biofilms attached to microtiter plates or silicone wafers were strained by crystal violet solution (Sigma-Aldrich, St Louis, MO) for 15 minutes. Excess stain was removed by washing with PBS. The crystal violet attached to biofilm samples was dissolved with ethanol. The absorbance at 595 nm was measured using SpectraMax® spectrophotometer (Molecule Probe, Sunnyvale, CA) as the value of biofilm formation. Percentage inhibition of sample treated with the small compound was calculated against the mean of samples treated with DMSO. Experiments were repeated three times to obtain the mean and standard error of means of biofilm formation under each treatment.

### Growth Curves

*S. aureus* RN6390 single colony was inoculated into THY media and cultured overnight at 37°C. Overnight culture was sub-cultured (1∶100) into test tubes with fresh THY plus 50 µM CCG-203592 or DMSO in triplicate. Optical density readings at 600 nm were obtained with SpectraMax® spectrophotometer (Molecule Probe, Sunnyvale, CA) over a period of 10 hours. Experiments were repeated three times to obtain the mean and standard error of means.

### Scanning Electron Microscopy

The biofilm formation of *S. aureus* RN6390 on silicone wafers was performed as described above. The biofilm samples on silicone wafers were fixed with 2% glutaraldehyde/2% paraformaldehyde in 0.1 M cacodylate buffer (pH 7.4) for 2 hours at 4°C, washed three times with 0.1 M cacodylate buffer (pH 7.4) and fixed with 0.1% osmium tetraoxide for 1 hour. The biofilm samples were washed with ultrapure distilled water and dehydrated by increasing concentrations of ethanol (20%, 50%, 70%, 90%, 95%, 100%, 100% and 100%) for 15 minutes each. The biofilm samples were coated by platinum sputter after critical-point drying and examined using a Quanta 600F scanning electron microscope (FEI Company, Hillsboro, OR). Experiment was performed in triplicate.

### Mammalian Cell Cytotoxicity Assay

Cell cytotoxicity assay was performed by following previous protocols [69]. HeLa cells were purchased from American Type Culture Collection (ATCC, Manassas, VA) and grown in Dulbecco’s modified Eagle’s medium (DMEM) (Life Technologies, Grand Island, NY) with 10% fetal bovine serum, 1% penicillin-streptomycin and 1% L-glutamine. Two hundred µl aliquots of cell suspensions (1.6×10^4^ per well) with different concentrations of CCG-203592 were plated in 96-well plates and grown for 24 hours at 37°C in 5% CO_2_ in quadruplicate. DMSO was used as the vehicle control. Twenty µl of CellTiter 96 AQueous One Solution reagent (Promega, Madison, WI) was added to each well and the plates were incubated for 2 hours. The absorbance at 490 nm was quantified with a SpectraMax® spectrophotometer (Molecule Probe, Sunnyvale, CA). One hundred percent viability was set at the absorbance of the cells treated with only the vehicle (DMSO). The assay was performed three times to obtain the mean and standard error of means of cell viability.

### Real Time RT-PCR

Overnight cultures of *S. aureus* RN6390 were diluted 1∶200 with fresh sterile THY medium containing 0.5% glucose. Four ml aliquots of the diluted cultures with 50 µM CCG-203592 or DMSO were added into culture tubes (Fisher Scientific Co., Pittsburgh, PA), and then cultured overnight at 37°C in triplicate. Samples were collected by centrifugation at OD_600 nm_∼0.5 (corresponding to ML growth phase), 0.9 (LL growth phase) and overnight (S) phase. Experiments were repeated three times. RNA in *S. aureus* cells was stabilized using RNAprotect Cell Reagent (Qiagen, Valencia, CA). *S. aureus* cells were digested by lysostaphin (Sigma, St. Louis, MO). RNA was then isolated by Trizol (Invitrogen, Carlsbad, CA) according to the manufacture’s protocol. After RNA extraction, TURBO DNA-free kit (Ambion, Austin, TX) was used to remove residual DNA contamination in the RNA samples. The purified RNA was reverse transcribed using iScript™cDNA Synthesis Kit (Bio-Rad, Richmond, CA). cDNA samples were quantified by real time PCR using CFX96 Real-Time PCR Detection System (Bio-Rad, Richmond, CA) and the 2^(−ΔΔCt)^ Method [70]. PCR primers for each tested genes were presented in Table 1. The expression levels of all selected genes were normalized using 16S rRNA as an internal standard [71].

### Statistical Analysis

Experimental data were analyzed with SigmaPlot 11.2 software (Systat Software Inc. Richmond, CA). Standard curves analysis was performed to generate dose-response curves and calculate IC_50_s. The results were presented as means±standard error of means (SDEM). Student’s t tests were performed. A p value of <0.05 was considered to be statistically significant.
